# Leaf Structural Traits Vary With Plant Size in Even-Aged Stands of *Sapindus mukorossi*

**DOI:** 10.3389/fpls.2021.692484

**Published:** 2021-07-22

**Authors:** Yunni Chang, Chaobin Xu, Hong Yang, Junxin Zhou, Weiping Hua, Shihe Zhang, Quanlin Zhong, Baoyin Li

**Affiliations:** ^1^Fujian Provincial Key Laboratory for Plant Eco-Physiology, Fujian Normal University, Fuzhou, China; ^2^College of Geographical Sciences, Fujian Normal University, Fuzhou, China; ^3^State Key Laboratory for Subtropical Mountain Ecology of the Ministry of Science and Technology and Fujian Province, Fujian Normal University, Fuzhou, China; ^4^College of Environmental Science and Engineering, Fujian Normal University, Fuzhou, China; ^5^Department of Geography and Environmental Science, University of Reading, Reading, United Kingdom; ^6^Department of Forestry, Fujian Forestry Vocational Technical College, Nanping, China; ^7^College of Ecological and Resources Engineering, Wuyi University, Wuyishan, China

**Keywords:** leaf structural traits, plant size, even-aged stands, *Sapindus mukorossi*, allometric relationships

## Abstract

*Sapindus mukorossi* Gaertn., an important oleaginous woody plant, has garnered increasing research attention owing to its potential as a source of renewable energy (biodiesel). Leaf structural traits are closely related to plant size, and they affect the fruit yield and oil quality. However, plant size factors that predominantly contribute to leaf structural traits remain unknown. Therefore, the purpose of this study was to understand the associations between leaf structural traits and plant size factors in even-aged stands of *S. mukorossi*. Results showed that leaf length (LL) and leaf area (LA) markedly increased with the increasing diameter at breast height (DBH) and tree height (TH), although other leaf structural traits did not show noticeable changes. Difference in slopes also indicated that the degree of effect of plant size factors on leaf structural traits was in the order of TH > DBH. Leaf structural traits showed no systematic variation with crown width (CW). LA was significantly positively correlated with LL, leaf width (LW), LL/LW, and leaf thickness (LT) and was significantly but negatively correlated with leaf tissue density (LTD) and leaf dry mass content (LDMC). Specific leaf area showed a significantly negative correlation with LT, LDMC, and LTD. LTD showed a significantly positive correlation with LDMC, but a negative correlation with LT. The results were critical to understand the variability of leaf structural traits with plant size, can provide a theoretical foundation for further study in the relationship between leaf structural traits and fruit yield, and regulate leaf traits through artificial management measures to promote plant growth and fruit yield.

## Introduction

Leaves are a vital component of the photosynthetic apparatus of a plant and play key roles in long-term adaptations to environmental changes (Liu et al., 2017; Guo et al., 2018; Song et al., 2018; Cai et al., 2020). Leaves are also critical for tree productivity due to their fundamental roles in carbon assimilation through photosynthesis and transpiration (Wright et al., 2004; Lai et al., 2016; Li et al., 2018). Leaf traits are currently gaining high research priority because they are closely linked to many pivotal aspects of growth, reproduction, and ecosystem functions (Garnier et al., 2001; Xue et al., 2012; Qin et al., 2019). Plant growth is driven by factors that affect resource (light, nutrients, and water) gain and utilization (Richards et al., 2010; Li et al., 2017). Leaf traits reflect both resource uptake strategies and resource use efficiency and are thus expected to affect plant growth (Fichtner et al., 2013; Li et al., 2017). Leaf traits as measurable characteristics represent ecological strategies and functional adaptations to environmental conditions (Pérez-harguindeguy et al., 2013; Petter et al., 2016; Qin et al., 2019; Bergholz et al., 2021). Key leaf structural traits include leaf length (LL), leaf width (LW), leaf thickness (LT), leaf area (LA), specific leaf area (SLA), and leaf dry mass content (LDMC), and others (Liu et al., 2006). Leaf structural traits reflect the resource acquisition under a long-term influence of external environment (Xue et al., 2012). LL, LW, and LL/LW are associated with leaf size and shape. LT responds in some way to the light capture and changes in the water status of leaf tissue (Seelig et al., 2015; Griffith et al., 2016). LA is a key variable for physiological studies involving plant growth, light interception, and photosynthetic efficiency (Kandiannan et al., 2009; Rouphael et al., 2010). SLA, as a measure of resource allocation, reflects the potential light capture per unit LA per unit organic matter content invested into leaves (Wright and Westoby, 1999). LDMC is a measure of leaf tissue density (LTD) and reflects the position of a plant on a resource use axis (Wilson et al., 1999). The associations among various leaf traits reflect the plant adaptation under a given constraint or other environmental constraints (Qin et al., 2019). Increasing evidence indicates that leaf traits are highly plastic at various growth stages (Thomas and Ickes, 1995). For example, in *Populus euphratica*, LT, LA, and LDMC showed a gradually increasing trend, and the SLA showed a decreasing trend with plant growth (Huang et al., 2010). In the same species, LL and LL/LW showed a decreasing trend and the LW showed an increasing trend with increasing tree age (Wang et al., 2019). In some evergreen trees, older leaves showed a significantly greater LA and LDMC but lower SLA than younger leaves (Huang et al., 2013). In *Betula platyphylla*, LT, SLA, and LDMC showed significant differences between adult trees and saplings at different growth stages (Jin et al., 2018).

Diameter at breast height (DBH) and tree height (TH) are the commonly used measures of tree growth (Sumida et al., 2013), and these are important variables used in forest inventories and management as well as in the forest carbon stock estimation (Li et al., 2015). Crown width (CW) is commonly used to estimate the aboveground biomass of forests and is often regarded as an important indicator of tree growth (Fichtner et al., 2013). These variables directly reflect the plant size and are used as plant size factors. Variation in leaf traits with plant size reflects plant life history and biomass allocation patterns (Huang et al., 2013). Liu et al. (2010) conducted a basic study of differences in leaf traits between small (woody plants shorter than 2 m) and large (woody plants taller than 10 m) trees based on TH. Their study indicated that SLA was higher in small trees than that in large trees (Liu et al., 2010). Along with the DBH growth, the LT, LA, and LDMC of *P. euphratica* showed a gradually increasing trend, while the SLA showed a decreasing trend (Huang et al., 2010). However, some studies showed that the size and functional traits of leaves are invariant with plant size (West et al., 1999). Most leaf functional traits are substantially less variable than plant size, show little to no systematic variation. However, individual LA itself increases modestly with plant size (Price et al., 2014). DBH is closely related to CW and TH (Dai et al., 2009; Li et al., 2015) because they both serve as general proxies for plant size. However, less is known regarding the specific associations between different plant size factors and leaf structural traits in even-aged forests, although this information may be critical to understand plant life history patterns (Liu et al., 2010).

*Sapindus mukorossi* Gaertn. is a deciduous oleaginous woody tree, which is widely distributed in the tropical and subtropical regions of Asia (Zhang et al., 2019). Its fruit pericarp contains saponin, which is an efficient natural surfactants used for developing high-quality shampoos and detergents (Bahar and Singh, 2007; Attri et al., 2015; Bhatta et al., 2021). The oil content of its seeds and kernels is high, and the seed oil meets the standards for biodiesel production, making it as a source of bioenergy (Zhao et al., 2014). Therefore, *S. mukorossi* is considered a valuable and promising industrial crop owing to the high content and quality of its seed oil (Zhang et al., 2019). Previous studies have mainly focused on *S. mukorossi* breeding, genetic diversity, biochemical analyses for identifying varieties that produce high seed oil and saponin yield (Shao et al., 2013; Diao et al., 2014; Sun et al., 2017), extraction techniques of saponins and its application in industry (Singh and Sharma, 2019a) and medicine (Huang et al., 2008; Li et al., 2013; Singh and Kumari, 2015; Singh and Sharma, 2019b; Zhao et al., 2021), and extraction techniques for seed kernel oil (Liu et al., 2019; Hu et al., 2021). Leaf size and shape can predict single fruit weight and other qualitative traits and can therefore be used as indicators for evaluating fruit quality (Jin et al., 2016; Long et al., 2017; Rowland et al., 2020). The saponins yield and oil content are important fruit traits. Studies have shown that the effect of plant size on the seed oil content of *S. mukorossi* was in the order of TH>DBH>CW, and the effect of plant size factor on the saponin content of *S. mukorossi* decreased in the order of DBH>TH>CW (Fan et al., 2013, 2014). The size of an oleaginous tree affects its fruit yield and oil quality to a certain extent (Wu et al., 2012; Guo et al., 2014). The inherent association between leaf traits and fruit characteristics has been explored in some previous studies (Wang et al., 2014; Zhang et al., 2020a). Therefore, leaf structural traits may also affect the fruit yield and oil quality of oleaginous trees. Although previous studies on leaf traits have laid a foundation for the selection of excellent fruit quality traits, no study has assessed the leaf structural traits of plants of different sizes in an even-aged forest in this species. In homogeneous even-aged bioenergy plantations, the plant size factors directly reflect the plant size and possibly indicate their effects on fruit yield and oil quality. In this study, we measured various leaf structural traits and assessed their associations with three plant size factors in *S. mukorossi*. The main objectives are to determine (1) whether there are notable correlations between leaf structural traits with plant size; (2) whether DBH, TH, and CW had consistent effects on leaf structural traits; and (3) which plant size factors directly reflect more leaf structural traits. By addressing these questions, we were able to understand the potentiality and sensibility of *S. mukorossi* leaf structural traits responding to plant size. Our findings are of great significance to provide a basis for studying the associations of leaf traits with fruit yield and quality, be helpful for predicting the yield to select superior individuals, and provide a theoretical basis for regulating plant growth and improving fruit yield with artificial management measures regulating the leaf traits of oleaginous trees.

## Materials and Methods

### Study Area

The field experiment was conducted in Nanping city, Yanping District (26°29′N, 118°13′E), northwest Fujian Province, southeast China. This region is characterized by a subtropical humid monsoon climate, with a mean annual temperature of 19.3°C, mean annual precipitation of 1,669 mm, and relative humidity of 82%. Summers are long, hot, and rainy, while winters are short and moderate. The soil in this zone is classified as red soil as per the Chinese Soil Taxonomic Classification (Fan et al., 2015). The initial total soil C, N, and P contents were 17.49, 1.12, and 0.45 g/kg, respectively. The *S. mukorossi* forest was established at an altitude of 120 m. The planting density was approximately 1,100 plants per hectare in 2013. Mean TW, DBH, and CW were 5.05 ± 1.26 m, 6.79 ± 2.53 cm, and 3.80 ± 1.04 m, respectively.

### Experimental Design

**Figure 1:**
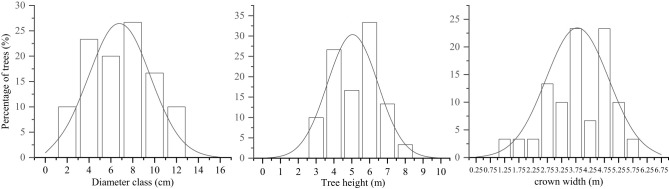
Distribution characteristics of *Sapindus mukorossi* trees with plant size factors.

This study was performed during the reproductive growth period during October 2019. Three 25.82 × 25.82 m sample plots were established, with a 10-m-wide buffer zone separating the plots. DBH (≥2 cm), CW, and TH were measured and recorded for all the trees. Ten trees were selected in each plot, and the distribution of plant characteristics was determined in 30 trees. The selected trees were categorized into individual size factor classes (DBH, CW, and TH) based on the upper limit exclusion method (Zhang et al., 2015a). The DBH classes were formed based on the second (1–2.9 cm), fourth (3–4.9 cm), sixth (5–6.9 cm), eighth (7–8.9 cm), tenth (9–10.9 cm), and twelfth (11–12.9 cm)-order diameters. The TH classes were formed based on the third (2.5–3.4 m), fourth (3.5–4.4 m), fifth (4.5–5.4 m), sixth (5.5–6.4 m), and seventh (6.5–7.4 m)-grade heights. The CW classes were formed based on the values at the 2.75th (<3 m), 3.25th (3–3.49 m), 3.75th (3.5–3.99 m), 4.25th (4–4.49 m), 4.75th (4.5–4.99 m), and 5.25th (>5 m) CW stages (Zhang et al., 2015a). The distribution of plant size values of the *S. mukorossi* forest was normal (Figure 1).

### Sampling and Data Collection

Pinnately compound leaves were collected from the east-, west-, north-, and south-facing sides of the middle-outer crown of each selected plant using a lopping machine. Eight fully expanded, mature, and healthy leaves were selected per plant and were taken to the laboratory. A single relatively complete, moderate-sized, and well-grown representative leaflet was selected for each pinnately compound leaf.

For each sample, leaf fresh weight (LFW, g) was measured immediately using an electronic balance. The LL (cm, the maximum value along the midrib), LW (cm, the maximum value perpendicular to the midrib), and LA (cm^2^) were measured by scanning the leaves (Epson Perfection V30) and analyzing the scans using the ImageJ software (National Institutes of Health, Bethesda, MD, United States). The LT was measured by using the Vernier calipers (precision of 0.01 mm) at three points on the upper, middle, and lower sides at an intermediate position between the leaf margin and midrib (avoiding the main vein), and the average LT value of each leaf was calculated. The leaves were then soaked in deionized water for over 12 h in a ziplock bag, and the leaf saturated fresh weight (LSFW, g) was measured after absorbing water from the surface. All leaves were oven-dried at 70°C for 48 h to a constant mass, and the leaf dry mass (LDM, g) was measured.

Leaf shape index, SLA, LDMC, LTD, and leaf relative water content (LRWC) were calculated using the following formulas (Zhou et al., 2017; Yu et al., 2018):

(1) $$\begin{array}{l} \text{Leaf shape index}=\text{LL}/\text{LW } \end{array}$$

(2) $$\begin{array}{l} \text{SLA }\left({\text{cm}}^{2}/\text{g}\right)=\text{LA}/\text{LDM } \end{array}$$

(3) $$\begin{array}{l} \text{LDMC }\left(\text{g}/\text{g}\right)=\text{LDM}/\text{LSFW } \end{array}$$

(4) $$\begin{array}{l} \text{LTD }\left(\text{g}/\text{c}{\text{m}}^{3}\right)=\text{LDM}/\left(\text{LA }\times \text{ LT}\right)\; \end{array}$$

(5) $$\begin{array}{l} \text{LRWC }\left(\%\right)=\left(\text{LFW }-\text{ LDM}\right)/\left(\text{LSFW }-\text{ LDM}\right)\\ \;\times 100\% \end{array}$$

where LL is the leaf length (cm), LW is the leaf width (cm), LA is the leaf area (cm^2^), LDM is the leaf dry mass (g), LSFW is the leaf saturated fresh weight (g), LFW is the leaf fresh weight (g), and LT is the leaf thickness (cm).

### Statistical Analysis

The relationships between leaf structural traits and plant size factors (e.g., DBH, TH, and CW) were analyzed by using Model II Standardized Major Axis (SMA) regression as follows:

(6) $$\begin{array}{l} \text{y}=\text{ax}{}^{b} \end{array}$$

To linearize the relationship, this formula was log-transformed to obtain the following equation:

(7) $$\begin{array}{l} \text{log}\left(\text{y}\right)=\text{log}\left(\text{a}\right)+\text{blog}\left(\text{x}\right)\; \end{array}$$

where *y* is the leaf structural traits, *x* refers to the plant size factor, *a* is the normalization constant (the intercept), and *b* is the scaling exponent (the slope). When *b* = 1, the equation describes an isometric relationship; when *b* ≠ 1, the equation describes an allometric relationship. Model Type II regression was used to determine the numerical values of *a* and *b* using the package “smatr” in R.3.6.1 (R Core Team, 2019). When the data among different groups showed no significant numerical differences for the scaling exponents (the slope, *b*), a common scaling exponent was calculated (Warton et al., 2006, 2012). The common slope was calculated from a pooled variance/covariance matrix. The significance level for testing slope heterogeneity was *p* < 0.05. In addition, the correlations among key leaf structural traits were examined by using Pearson's correlation analysis. Pearson's correlation coefficients for all leaf structural traits were calculated using the “corrplot” package in R.3.6.1.

## Results

### Leaf Structural Traits and Plant Size

**Figure 2:**
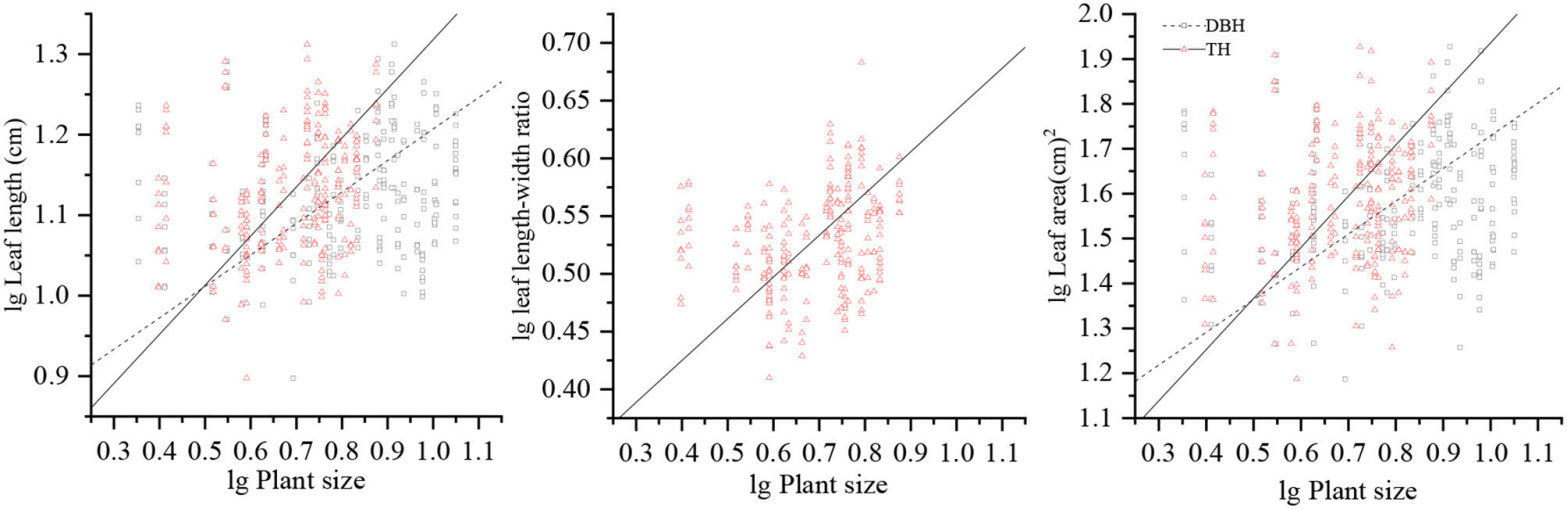
Allometric relationships of LL, LL/LW, and LA with respect to DBH and TH. LL, leaf length; LW, leaf width; LL/LW, leaf length–leaf width ratio; LA, leaf area; DBH, diameter at breast height; TH, tree height.

LL and LA were significantly and positively correlated with DBH (*p* < 0.05), while there were no significant correlation between the LW, LL/LW, LT, SLA, LDMC, LTD, and LRWC with DBH (*p* > 0.05). LL, LL/LW, and LA showed a modest increase with TH (*p* < 0.05), and there were no significant correlation between LW, LT, SLA, LDMC, LTD, and LRWC with TH (*p* > 0.05); these relationships showed low *R*^2^ values. The steeper slopes of the relationships between LL, LL/LW, and LA with TH and with DBH indicate that LL, LL/LW, and LA increase more rapidly with TH than those with DBH. All leaf structural traits approach invariance with CW, indicating that it is not associated noticeably with leaf structural traits (Table 1 and Figure 2).

**Table 1:** Allometric relationships between leaf structural traits and plant size factors.

***Y*-variable**	***X*-variable**	***R^**2**^***	***p***	**Slope**	**95% CI**	**Intercept**	**95% CI**
LL	DBH	0.024	**0.016**	0.391	0.345, 0.443	0.816	0.775, 0.857
LW	DBH	0.008	0.158	0.363	0.320, 0.412	0.310	0.271, 0.348
LL/LW	DBH	0.014	0.066	0.232	0.204, 0.263	0.344	0.319, 0.368
LT	DBH	<0.001	0.748	−0.339	−0.339, −0.385	−0.546	−0.582, −0.509
LA	DBH	0.021	**0.024**	0.730	0.644, 0.828	0.999	0.922, 1.076
SLA	DBH	<0.001	0.853	−0.309	−0.350, −0.272	2.467	2.434. 2.499
LDMC	DBH	<0.001	0.974	−0.178	−0.202, −0.157	−0.296	−0.315, −0.277
LTD	DBH	0.001	0.593	0.311	0.274, 0.353	−0.653	−0.686, −0.620
LRWC	DBH	0.002	0.531	0.225	0.198, 0.255	−0.279	−0.303, −0.256
LL	TH	0.063	** <0.001**	0.610	0.539, 0.690	0.708	0.654, 0.761
LW	TH	0.012	0.092	0.566	0.499, 0.643	0.209	0.158, 0.260
LL/LW	TH	0.063	** <0.001**	0.362	0.320, 0.409	0.280	0.248, 0311
LT	TH	0.002	0.539	0.528	0.465, 0.600	−1.179	−1.226, −1.131
LA	TH	0.044	**0.001**	1.139	1.005, 1.290	0.797	0.697, 0.897
SLA	TH	<0.001	0.673	−0.481	−0.546, −0.424	2.552	2.509, 2.596
LDMC	TH	0.001	0.622	0.278	0.244, 0.315	−0.629	−0.654, −0.604
LTD	TH	<0.001	0.801	−0.485	−0.550, −0.427	−0.072	−0.116, −0.029
LRWC	TH	0.006	0.215	−0.351	−0.398, −0.309	0.141	0.109, 0.172
LL	CW	0.015	0.056	0.513	0.452, 0.582	0.840	0.802, 0.879
LW	CW	0.005	0.285	0.476	0.419, 0.541	0.332	0.296, 0.368
LL/LW	CW	<0.001	0.978	1.028	0.905, 1.168	1.320	1.242, 1.399
LT	CW	0.002	0.464	−0.444	−0.504, −0.391	−0.566	−0.600, −0.533
LA	CW	0.014	0.068	0.957	0.843, 1.086	1.044	0.972, 1.116
SLA	CW	<0.001	0.918	0.405	0.356, 0.460	1.995	1.964, 2.025
LDMC	CW	0.003	0.415	−0.233	−0.265, −0.206	−0.307	−0.325, −0.289
LTD	CW	0.002	0.486	0.407	0.359, 0.463	−0.634	−0.665, −0.603
LRWC	CW	<0.001	0.831	−0.295	−0.335, −0.259	0.064	0.042, 0.087

*LL, leaf length; LW, leaf width; LL/LW, leaf length–leaf width ratio; LT, leaf thickness; LA, leaf area; SLA, specific leaf area; LDMC, leaf dry mass content; LTD, leaf tissue density; LRWC, leaf relative water content; TH, tree height; CW, crown width; DBH, diameter at breast height. Values in bold indicate significance at P < 0.05*.

### Associations Among Leaf Structural Traits

**Figure 3:**
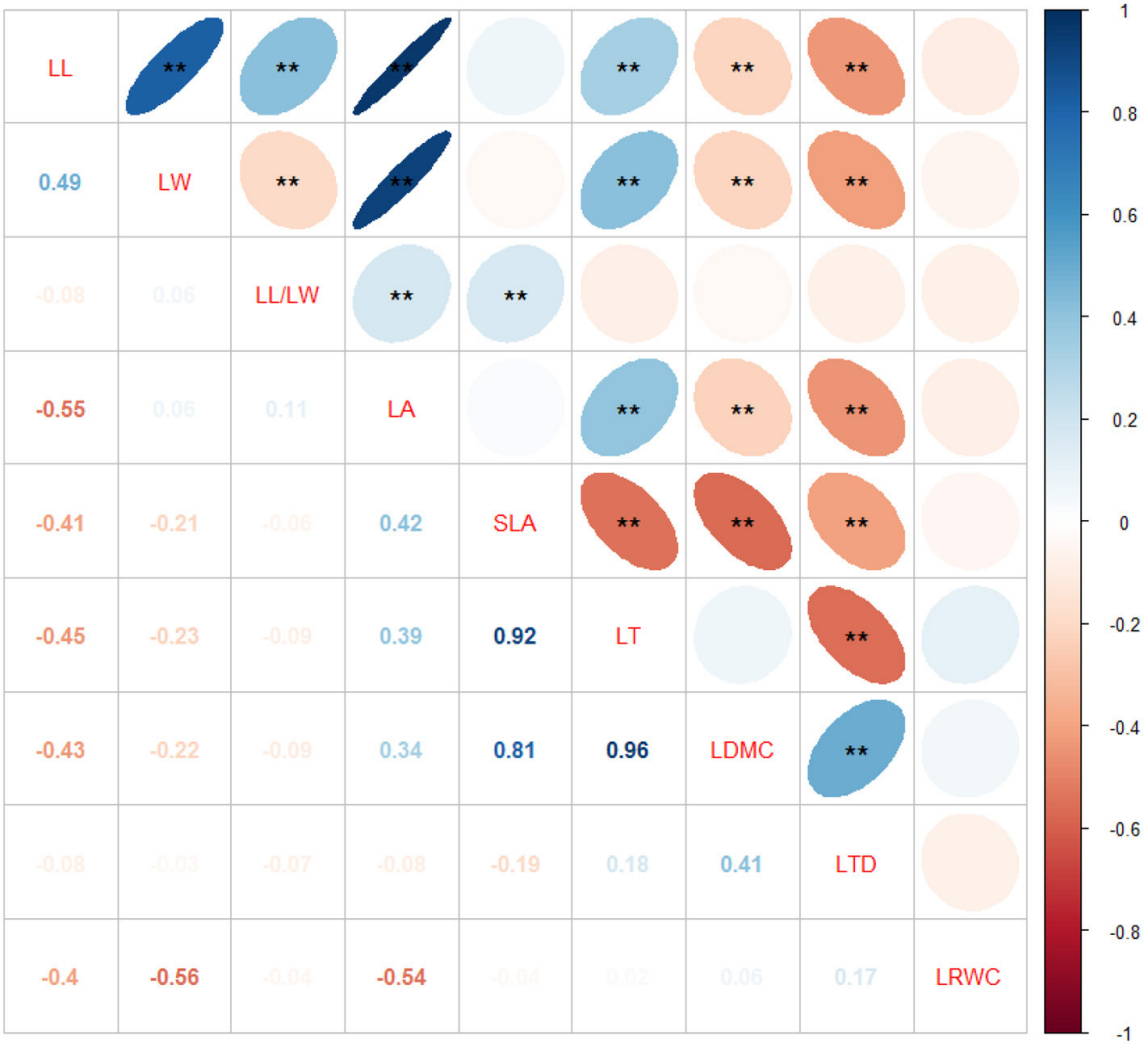
Correlations among leaf structural traits of *Sapindus mukorossi*. Correlation coefficients of the blue and red ellipses are positive and negative, respectively. Darker the color and smaller the area of the ellipse, greater the degree of correlation, and larger the absolute value of correlation coefficient. Variable names are on the diagonal. **p < 0.01.

Leaf traits are not independent of each other; therefore, correlations among leaf structural traits have been observed. LL was significantly and positively correlated with LW, LL/LW, LT, and LA and significantly but negatively correlated with LDMC and LTD. LW was significantly and positively correlated with LA and LT and significantly but negatively correlated with LL/LW, LDMC, and LTD. LL/LW was significantly and positively correlated with LA and SLA. LA was positively correlated with LT but negatively correlated with LDMC and LTD. SLA was significantly but negatively correlated with LT, LDMC, and LTD. LT was negatively correlated with LTD. LDMC was positively correlated with LTD (Figure 3).

## Discussion

### Effects of TH, DBH, and CW on Leaf Structural Traits

Leaf traits may reflect adaptations to environmental variations and can thus elucidate the associations between environmental drivers and ecosystem functions (Guo et al., 2020). Leaf traits are significantly correlated with plant growth and development. Previous studies indicated that the pine nut yield had a linear correlation with plant crown size (Zhang et al., 2015b), and the inherent association between leaf traits and fruit characteristics has also been found. Assessing the leaf structure characteristics of oleaginous trees of different sizes can help to better regulate leaf traits and improve fruit yield. In general, LL, LW, LL/LW, and LA can accurately reflect the leaf shape and size, and changes, which may represent adaptive strategies of plants to the changing environments. Collectively, our results suggest that LL, LL/LW, and LA vary as a function of TH or DBH, and this is consistent with previous evaluations (Fonseca et al., 2000; Wright et al., 2007; Price et al., 2014). The variability in LL in short trees reflects the adaptability of leaves to extrinsic micro- and macroenvironmental factors and stresses (Jensen and Zwieniecki, 2013). Jensen and Zwieniecki (2013) focused only on trees taller than 20 m and proposed that the factors limiting leaf size can be understood by subjecting the plants to physical constraints imposed by intrinsic (biological and geometrical) properties of the carbohydrate transport network. The trees included in our study were not large enough (shorter than 10 m), so without the restriction of water potential transport, longer sieve tubes of leaves enable somewhat greater transport efficiency and better nutrient uptake. Meanwhile, LA can change the photosynthetic capacity by affecting the optical light interception efficiency, and plants can increase the total photosynthetic area by increasing LA to promote plant growth. However, the positive correlation between leaf size and plant size was reversed in other studies (Price et al., 2014; Wang et al., 2019). A possible explanation for the reversed trend may be the greater support and hydraulic costs of larger leaves (Price et al., 2014). In *P. euphratica*, LL and LL/LW decreased with plant growth (Wang et al., 2019), and changes in leaf shape may be an adaptive strategy to the desert environments. Another reason for the different result may be the variation in tree species or habitats. We observed a decrease in the amount of variance for each functional trait with DBH than that with TH. Difference in slopes indicated that the effects of plant size factors on leaf structural traits were in the order of TH > DBH. As TH increases, the volume and depth of soil explored by the root system also increase; therefore, taller trees can take up more nutrients to supply to their leaves (Liu et al., 2010). Some studies have shown that the root system of taller plants is more competitive and can acquire more resources (Thomas and Winner, 2002). They also showed greater LA to enhance optical radiation absorption, improve photosynthesis, and increase biomass for survival and growth. However, shorter trees usually have shallower roots and lower trunk runoff; therefore, they cannot acquire sufficient light and water (Ou and Liu, 2017). The leaf structural traits that we evaluated showed no systematic variation with CW. The major reason may be that leaf structural traits were linked to canopy thickness, permeability of the canopy, and leaf area index (Lü et al., 2007). Moreover, CW was in close relation with planting density, indicating that planting density has no significant influences on leaf structural traits (Gong et al., 2010).

The leaf thickness reflects plant resource acquisition and water conservation. SLA can be used as an indicator of the carbon acquisition strategy as well as of the ability of the plant to acquire resources, such as light, and protect itself under strong light (Zhang et al., 2020b). LDMC can be used to characterize the ability of the plant to conserve nutrients and resist physical damage (Zhang et al., 2016). LTD is related to plant drought tolerance and damage resistance (Guo et al., 2020). LRWC is closely related to photosynthesis and water use efficiency, and the photosynthetic rate could be improved by reducing LRWC (Duan et al., 2017). These traits did not change significantly with plant size. These trends are contrary to those reported in other studies (Huang et al., 2010; Zhang et al., 2020b). For *P. euphratica*, the LT and LDMC showed a gradually increasing trend and the SLA showed a decreasing trend with plant growth (Huang et al., 2010; Wang et al., 2019). Increases in LT, developed palisade tissue, cuticle thickness, and a number of mucus cells reduce water loss in plants to a certain extent by decreasing the transpiration through leaves and improving the water retention capacity (Huang et al., 2010). In a previous study in a tropical montane rainforest, the SLA of 127 small individuals (woody plants shorter than 2 m) and 47 large trees (woody plants taller than 10 m) was measured; SLA was higher in small individuals than that in large trees (Liu et al., 2010). A small SLA in large individuals can be attributed to their responses to the changing light competition and water conditions. Our results may also be because of the difference in survival environment; *S. mukorossi* is distributed in the tropical and subtropical regions, indicating the absence of water scarcity. Another possible explanation is the inclusion of different tree species in various studies.

**Figure 4:**
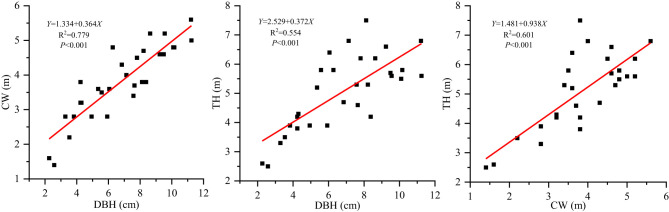
Correlation analysis among plant size indicators of *Sapindus mukorossi*.

Overall, there were no definite trends of changes in leaf structural traits with different plant size factors, except for the positive correlations of TH and DBH with LL, LL/LW, and LA. CW also has little explanatory power with respect to leaf structural traits. Looking at the variance also suggests that the leaf traits we examined displayed consistently low variance, with shallow slopes and low *R*^2^ values. This may be explained by several reasons. First, *S. mukorossi* is a deciduous tree, and the collected leaves were current-year leaves of even-aged stands. Studies have found that the leaf shape index and water content did not differ significantly between the small and large current-year fruiting twigs in *Morus alba L*. (Wang et al., 2014). Moreover, since the leaves were selected during the same reproductive growth period, the photosynthetic products were gradually shifted to the reproductive organs to ensure their growth for normal flowering and fruiting, and the leaf growth became stagnant (Zhang et al., 2016). In our study, DBH was significantly and positively correlated with CW and TH (Figure 4), and an increase in the DBH and TH increased the total biomass. Some biomass estimation models based on CW, DBH, and TH have also been established (Xu et al., 2013; Dong et al., 2015). Therefore, individual biomass likely increased with plant growth, although leaf traits did not change significantly with increasing DBH, TH, and CW. As the leaf biomass increases, LA becomes larger and more light can be received, and as the light energy utilization becomes more efficient, more photosynthetic products can be accumulated.

### Correlations Among Leaf Structural Traits

Leaf structural traits do not function independently and are correlated with one another. The relationships among leaf traits are closely linked to plant life history patterns. LL, LW, and LL/LW can accurately reflect the leaf shape. LA represents the contact area between leaves and the external environment, and this trait is affected by the balance of gas and energy exchange between the plants and the atmosphere. In this study, LA was positively correlated with LL, LW, LL/LW, and LT and negatively correlated with LTD and LDMC. In general, plants with a larger LA have thicker leaves, which prevents the heat exchange between leaves and the surrounding air and slows the diffusion rates of water vapor and CO_2_ through the leaves. Many studies have reported a positive correlation between LA and LT, but trends of association of LA with LDMC, LTD, and LRWC vary across studies (Zhang et al., 2016; Duan et al., 2017; Yu et al., 2018). These results indicate that correlations among leaf traits vary across research scales, plant species, and habitats.

SLA and LDMC are important leaf traits, which can reflect the survival strategies of plants for adapting to changing environments. Our study showed that SLA was significantly but negatively correlated with LT, LDMC, and LTD. These trends are consistent with previous reports (Huang et al., 2010; Guo et al., 2020) and indicate that *S. mukorossi* balances different leaf functional traits to adapt to the environment. Plants with a smaller SLA usually possess leaves with a greater LT (Reich et al., 1998). LT reflects the strategies of the plant for acquiring and utilizing resources. The thinner the leaves, the faster the plant growth and the stronger the light interception ability. For plants with a smaller SLA, a larger part of the materials in leaves is used to construct a protective structure or increase the density of mesophyll cells; thus, these plants often possess thicker leaves (Huang et al., 2010). From the perspectives of survival strategies and energy required to develop organs, if the plants use more energy to build the defense structures of the leaves, there is no additional energy to increase LA (Huang et al., 2010). Therefore, there was no significant correlation observed between LA and SLA in this study. However, many studies have reported a significant positive or negative correlation between LA and SLA (Zhang et al., 2016; Huang et al., 2020). LDMC reflects LTD as well as ecological functions and resource acquisition of plants (Dao et al., 2016). Our data demonstrated that LDMC was positively correlated with LTD (Cornelissen et al., 2003). Increased LDMC decreases the leaf water content, which in turn increases LTD. Increased LTD reduces the light transmission and decreases the photosynthetic capacity, which further reduces SLA. These results are consistent with the reports of Wang et al. (2015). LTD was significantly but negatively correlated with LT and LA, indicating that increases in LTD decreased LT and LA, thereby increasing LDMC. LRWC is closely linked to photosynthesis, and reduced LRWC can improve the water use efficiency and photosynthesis (Duan et al., 2017). In this study, the LRWC was not significantly correlated with any leaf traits. This may be because *S. mukorossi* grows in subtropical humid regions, where water is not a limiting factor.

The study has some potential limitations that should be addressed in future research. Leaf traits may exhibit considerable plasticity in response to environmental changes. Waigwa et al. (2020) found that LT had a positive correlation with soil phosphorous, and SLA had a strong negative relationship with soil total nitrogen. Morphological traits of tropical evergreen oaks were also correlated with the mean annual temperature, mean annual precipitation sum, and soil pH (Lin et al., 2021). However, environmental parameters were unavailable in this study. Meanwhile, leaf nutrient traits are also key functional traits, and focusing only on leaf structural traits might provide limited information. Therefore, the relationships between leaf traits (leaf structural traits and leaf nutrient traits) and environmental parameters (especially rhizosphere soil properties, temperature, and precipitation) should receive more attention in future research. In addition, leaf traits can predict fruit traits, and therefore, they can be used as indicators for evaluating fruit quality (Rowland et al., 2020). In hybrid pear, LA was significantly correlated with fruit vertical diameter and fruit type (Zhang et al., 2009). In Chinese bayberry, LL, LW, and LT played important roles in establishing the acid–sugar (AS) ratio (Liang et al., 2010). Further research is also warranted into the inherent association between leaf traits and fruit characteristics of *S. mukorossi*.

## Conclusions

A key result of our study is that LL, LL/LW, and LA of the oleaginous deciduous broad-leaved woody tree *S. mukorossi* increased with increasing DBH and TH. Furthermore, TH has more explanatory with respect to leaf traits than DBH. TH was the key plant size factor affecting the leaf structural traits of *S. mukorossi*. All leaf structural traits did not vary significantly with increasing CW. LA was positively correlated with LL, LW, LL/LW, and LT and negatively correlated with LDMC and LTD. SLA showed a significantly negative correlation with LT, LDMC, and LTD. LDMC was positively correlated with LTD. The covariation indicated that *S. mukorossi* adjusted and balanced the combination of leaf structural traits in response to environmental changes. Considering that climate, soil, and topographic variables can influence leaf traits, future progress requires more research on the effects of environmental factors on leaf traits. The influence of leaf traits on fruit characteristics also differs among various species. Further studies are needed to investigate the inherent association between leaf traits and fruit characteristics of *S. mukorossi* to predict and increase fruit yield.

## Data Availability Statement

The original contributions presented in the study are included in the article/supplementary material, further inquiries can be directed to the corresponding authors.

## Author Contributions

YC: Data curation, investigation, methodology, software, visualization, and writing (original draft). CX: Conceptualization, investigation, methodology, visualization, and writing (review and editing). HY: Conceptualization and writing (review and editing). JZ: Conceptualization, data curation, and investigation. WH: Software and visualization. SZ: Data curation and investigation. QZ: Conceptualization, funding acquisition, investigation, and writing (review and editing). BL: Conceptualization, methodology, supervision, validation, and writing (review and editing). All authors contributed to the article and approved the submitted version.

## Conflict of Interest

The authors declare that the research was conducted in the absence of any commercial or financial relationships that could be construed as a potential conflict of interest.
